# New-Onset Atrial Fibrillation in Patients with Pacemakers and the Implications of Hepatic Impairment

**DOI:** 10.3390/life15030450

**Published:** 2025-03-13

**Authors:** Adrian-Ionuț Ungureanu, Georgică Târtea, Anca Oana Docea, Cristina Elena Negroiu, Cristina Maria Marginean, Radu Mitruț, Marina-Carmen Deutsch, Eugen Țieranu, Radu-Gabriel Vătășescu, Paul Mitruț

**Affiliations:** 1 Doctoral School, University of Medicine and Pharmacy of Craiova, 200349 Craiova, Romania; adrianungureanu90@yahoo.com; 2 Department of Cardiology, Emergency County Hospital of Craiova, 200642 Craiova, Romania; eugen.tieranu@umfcv.ro; 3 Department of Physiology, University of Medicine and Pharmacy of Craiova, 200349 Craiova, Romania; 4 Department of Toxicology, University of Medicine and Pharmacy of Craiova, 200349 Craiova, Romania; ancadocea@gmail.com; 5 Department of Pathophysiology, University of Medicine and Pharmacy of Craiova, 200349 Craiova, Romania; cristina.negroiu@yahoo.ro; 6 Department of Internal Medicine, University of Medicine and Pharmacy of Craiova, 200349 Craiova, Romania; marginean22@yahoo.com (C.M.M.); paulmitrut@yahoo.com (P.M.); 7 Department of Cardiology, University and Emergency Hospital, 050098 Bucharest, Romania; radu-mitrut@yahoo.co.uk; 8 Department of Cardiology, Colentina Clinical Hospital, 020125 Bucharest, Romania; carmenmarinailie@gmail.com; 9 Department of Cardiology, University of Medicine and Pharmacy of Craiova, 200349 Craiova, Romania; 10 Department of Cardiology, Faculty of Medicine, Carol Davila University of Medicine and Pharmacy, 050474 Bucharest, Romania

**Keywords:** dual-chamber pacemaker, atrial fibrillation detection, hepatic impairment

## Abstract

(1) Background: Atrial fibrillation (A Fib) is a common arrhythmia that affects millions of people worldwide and is characterized by irregular and often rapid heartbeats that can cause stroke. The aim of our study was to assess the importance of predictors for the occurrence of atrial fibrillation in patients with cardiac pacemakers and to analyze their impact on these patients, especially the impact of hepatic impairment. (2) Methods: This study is an observational, retrospective study, including 182 patients who were implanted with a dual-chamber pacemaker (DDD), with no known history of A Fib. (3) Results: We identified as predictors for the occurrence of atrial fibrillation in patients with cardiac pacemakers, DDD with rate response mode, NYHA class III of heart failure, as well as the presence of hepatic impairment (HI). Analysis of echocardiographic parameters of the left atrium revealed a larger left atrial volume as well as a larger left atrial area compared to patients who had a much smaller area at baseline and who did not experience any atrial fibrillation at follow-up. The fact that there were no statistically significant differences between the two groups of patients in terms of left atrial ejection fraction at baseline was very interesting. Patients in the A Fib group had a higher percentage of atrial pacing at the 9-month follow-up (86.23 ± 22.19%) compared to patients in the group without A Fib (44.92 ± 29.99%, *p* < 0.0001) and had a 9-month follow-up rate of A Fib of 25.806% vs. 2.247% in those with a low percentage of atrial pacing (*p* < 0.0001). The percentage of ventricular pacing at the 9-month follow-up, the observations were almost similar. (4) Conclusions: The importance of pacemakers in detecting subclinical episodes of atrial fibrillation remains crucial for the prevention of embolic events in these patients. Hepatic impairment may be a risk factor for the occurrence of atrial fibrillation in patients with pacemakers, but it can also create significant problems in stroke prevention.

## 1. Introduction

Atrial fibrillation (A Fib) is a common arrhythmia that affects millions of people worldwide, and it is characterized by an irregular and often rapid heartbeat [1].

The prevalence of A Fib increases with age, and between 2010 and 2019, the global prevalence of atrial fibrillation is estimated to have increased from 33 million to approximately 59 million patients living with atrial fibrillation [2,3]. Although A Fib can be asymptomatic, it can lead to various complications, including stroke, heart failure, and other cardiovascular diseases [4]. Paroxysmal atrial fibrillation (PA Fib) is a type of A Fib that is typically characterized by brief episodes lasting less than 7 days and ending spontaneously [1]. PA Fib is often asymptomatic and, as a result, may remain undetected for a long time. However, PA Fib increases the risk of stroke and heart failure, making early detection and treatment crucial to reduce mortality and the burden of A Fib [1,4].

Cardiac pacemakers (cardiac implantable electronic devices—CIEDs) are commonly used to manage various types of cardiac arrhythmias. They are designed to monitor and regulate the heart rhythm and can be programmed to detect and record A Fib episodes [5].

The prevalence of A Fib in patients with pacemakers varies considerably in the literature. Studies have reported prevalence rates of A Fib ranging from 10% to 50% in patients with pacemakers [6,7,8]. The variation in reported prevalence rates may be due to differences in patient populations, pacemaker programming, and duration of follow-up. However, it is clear that A Fib is a common finding in patients with pacemakers and should be considered in the management of these patients [9].

Diagnosing A Fib in patients with pacemakers can be challenging because many episodes of A Fib are asymptomatic and may not be detected without appropriate pacemaker programming [10]. Pacemakers can be programmed to detect A Fib episodes based on a variety of criteria, including episode duration, ventricular rate during the episode, and timing of the episode relative to other cardiac events. Recent research has used artificial intelligence in discriminating A Fib in patients with CIED [11]. In addition, some pacemakers are equipped with algorithms that can distinguish A Fib from other types of arrhythmias, such as supraventricular tachycardia [12]. When an A Fib episode is detected, the pacemaker can store the data for later review by a physician or transmit the data wirelessly to a monitoring center for real-time review.

Management of A Fib in patients with pacemakers is similar to that of A Fib in patients without pacemakers. Management goals include stroke prevention, rhythm control, and heart rate control to reduce symptoms and to improve overall cardiac function.

A new direction of research in recent years has targeted the possible relationship between A Fib and liver disease [13,14,15,16,17,18]. The relationship is intricate and influenced by multiple factors, including inflammation, oxidative stress, and structural remodeling of the atria, all in response to advancing liver disease.

The aim of our study was to evaluate the predictors of atrial fibrillation in a group of patients with cardiac implantable electronic devices and to analyze the impact of impaired liver function on them.

## 2. Materials and Methods

### 2.1. Study Design

This study is an observational, retrospective, cohort-based study that ultimately included 182 patients who were implanted with a dual-chamber pacemaker, with no known history of atrial fibrillation.

To avoid bias, all patients were consecutively included between October 2020 and January 2023. The aim was to assess the occurrence and identify predictors related to the analyzed outcome: the onset of atrial fibrillation detected by pacemaker devices and correlated with risk factors. The research followed the principles enshrined in the Declaration of Helsinki and received approval from the Ethics Committee of the University of Medicine and Pharmacy of Craiova, with reference to opinion number 108/1 March 2024.

### 2.2. Sample and Data Collection

Patients enrolled in this study received either the St. Jude Endurity or Medtronic/Vitatron devices. These pacemakers were equipped with advanced diagnostic features specifically designed for automated A Fib monitoring.

Patients were divided into two major groups (Appendix A) based on pacing mode:

1. DDDR pacing mode was programmed in patients who had a history or evidence of sinus node dysfunction (tachycardia-bradycardia syndrome, chronotropic incompetence, sinus pauses) but without a history of A Fib/A flutter.

2. DDD pacing mode in patients with AV conduction disorders and no history of the above-mentioned rhythm disorders.

**Figure 1:**
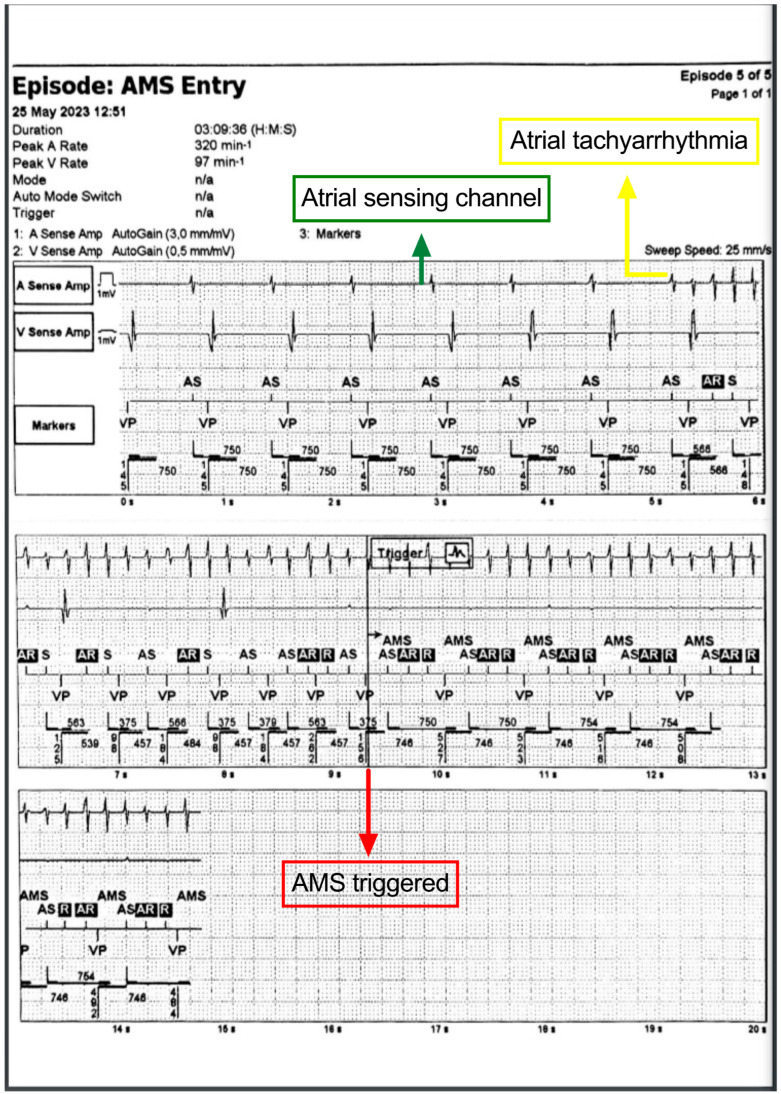
Image showing atrial fibrillation/flutter detected by St. Jude pacemaker, which triggers “automatic mode switch” (AMS) pacing mode (asynchronous DDI pacing mode).

The study began before the time of implantation, and the first visit was usually at least one month after pacemaker implantation, allowing sufficient time for stabilization and maturation of the electrodes. The minimum follow-up time was 9 months. Patients who were diagnosed with PA Fib either on a standard ECG or with AHR events considered atrial fibrillation/flutter and received chronic oral anticoagulant therapy based on the CHADs-VASc score. Stored pacemaker data were retrieved and analyzed using only PA Fib episodes that exceeded 5 min at periodic check-ups (Figure 1).

Patients with a history of paroxysmal atrial fibrillation/atrial flutter, patients with a single-chamber pacemaker, and patients with secondary hepatic damage were excluded from the study (see also Section 2.3. Hepatic Disease).

Patients subsequently had baseline follow-up visits at one month, three months, and six months, during which A Fib-related data were uploaded and analyzed. This included maintaining a diary of A Fib episodes, capturing electrograms (EGMs) and markers associated with stored episodes, recording the number and duration of episodes, and measuring heart rate before and at the onset of the arrhythmia. These regular follow-up visits were intended to monitor the progression and characteristics of A Fib in each patient.

**Figure 2:**
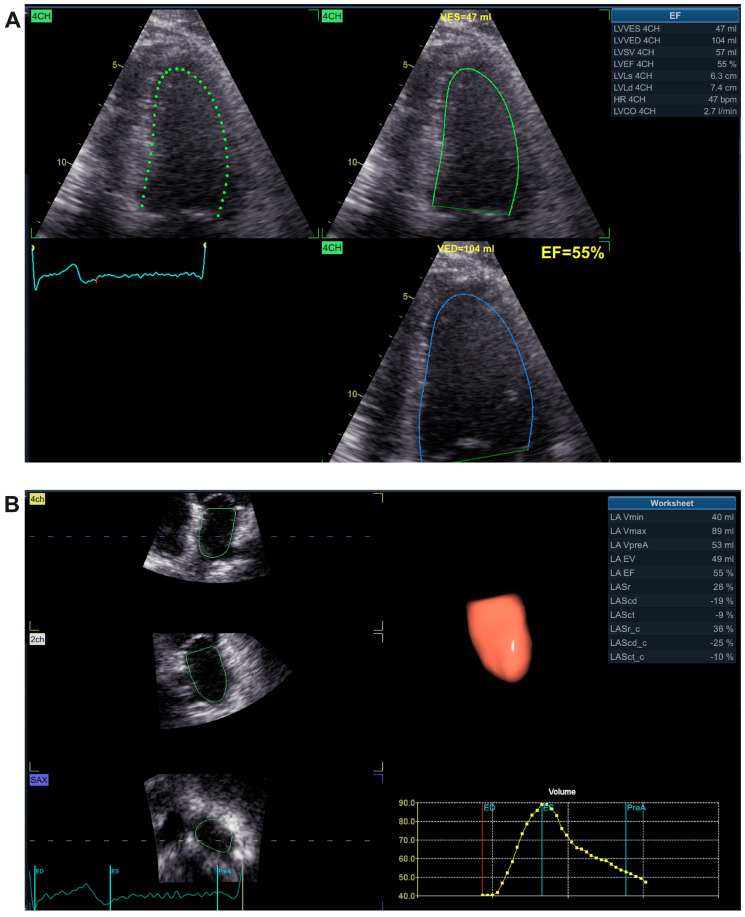
(**A**) Example of determining the left ventricular ejection fraction (LVEF). (**B**) Example of assessing the parameters of the left atrium: volume and ejection fraction.

Echocardiographic assessment was performed using the GE VIVID E9 XD Clear and PHILIPS AFFINITI 70 echocardiographs with standard software for measuring baseline parameters, valvular disease, and systolic function. Among the main echocardiographic parameters, left ventricular ejection fraction (LVEF) or left atrial parameters (volume, area, and ejection fraction) were monitored and included in our analysis (Figure 2).

### 2.3. Hepatic Disease

Hepatic impairment will be defined by an increase in transaminases above the normal value (mild HI) and >3x the normal limit value (major HI) and/or an increase in spontaneous INR > 1.5, under the exclusion of other secondary causes of hepatic impairment (viruses and other liver infections, primary liver diseases, etc.). The main cause of the increase in liver cytolysis markers in the patients included in this study will be identified as part of a cardio-hepatic syndrome, triggered by liver stasis in the context of cardiac disorders, such as rhythm disorders, valvulopathies, or ventricular systolic/diastolic dysfunction.

### 2.4. Statistical Analysis

Continuous variables were summarized as mean ± standard deviation (SD) or median and range for normally distributed and skewed data, respectively. Normality of continuous variables was assessed using the Kolmogorov–Smirnov test. Between-group comparisons for continuous variables were performed using either the *t*-test or the Mann–Whitney U-test. Categorical variables were presented as frequencies and percentages. The log-rank test was used to assess the occurrence of atrial fibrillation at follow-up. All statistical analyses were performed using GraphPad software, version 10.0 (La Jolla, CA, USA). A significance level of *p* < 0.05 was considered statistically significant.

## 3. Results

Our study ultimately included 182 patients who received a dual-chamber pacemaker between October 2020 and January 2023, without a history of atrial fibrillation. The main characteristics at baseline, before pacemaker implantation, of the patients included in our study are shown in Table 1. These characteristics are shown according to the end point of our study, the presence or absence of atrial fibrillation at follow-up.

We observed that the occurrence of atrial fibrillation in patients who received a pacemaker was not influenced by gender, there being no statistically significant difference. However, the age of the patients had an impact on the patients in the sense that patients with an older age (77.31 ± 7.09 years) presented a higher proportion of atrial fibrillation in the post-implant period compared to patients with a younger age (*p* = 0.0038). Analyzing the left ventricular ejection fraction (LVEF) for the patients included in our study, at baseline, no difference was observed between this parameter in patients who subsequently developed atrial fibrillation versus patients who did not develop atrial fibrillation. The same observation was also evident in the analysis of arterial hypertension and QRS complex duration. On the other hand, the group of patients with rate-response pacing mode activated recorded a higher incidence of atrial fibrillation (27%) compared to patients in whom rate-response mode activation was not required. Moreover, patients with impaired liver function recorded a higher incidence of atrial fibrillation compared to patients who did not have impaired liver function (35.29% vs. 9.46%, *p* = 0.0004).

**Figure 3:**
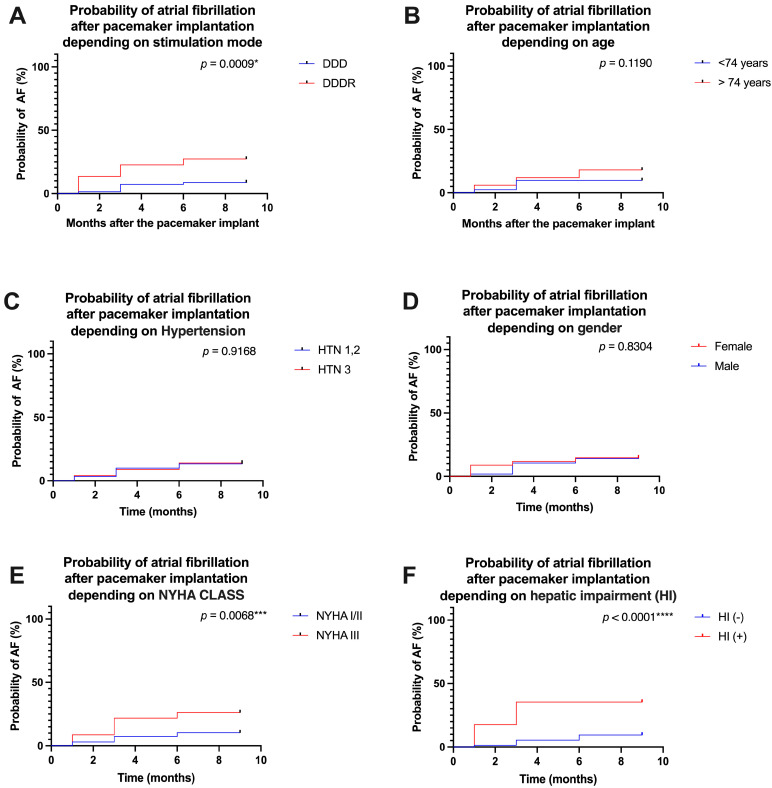
Probability of atrial fibrillation after pacemaker implantation depending on stimulation mode (**A**), age (**B**), hypertension (**C**), gender (**D**), NYHA Class (**E**), and hepatic impairment (**F**). **** *p* < 0.0001 *** *p* < 0.001; * *p* < 0.05.

Analyzing the probability of atrial fibrillation occurrence according to the parameters analyzed above, we observed that the rate response stimulation mode represented a strong predictor for the occurrence of atrial fibrillation. Patients with chronotropic incompetence, therefore requiring rate response activation, had a rate of A Fib occurrence at the 9-month follow-up of 27.272% vs. without A Fib, where the rate was only 8.695% (HR = 3.449, CI 1.323 to 8.994, *p* = 0.0009, Figure 3A).

**Figure 4:**
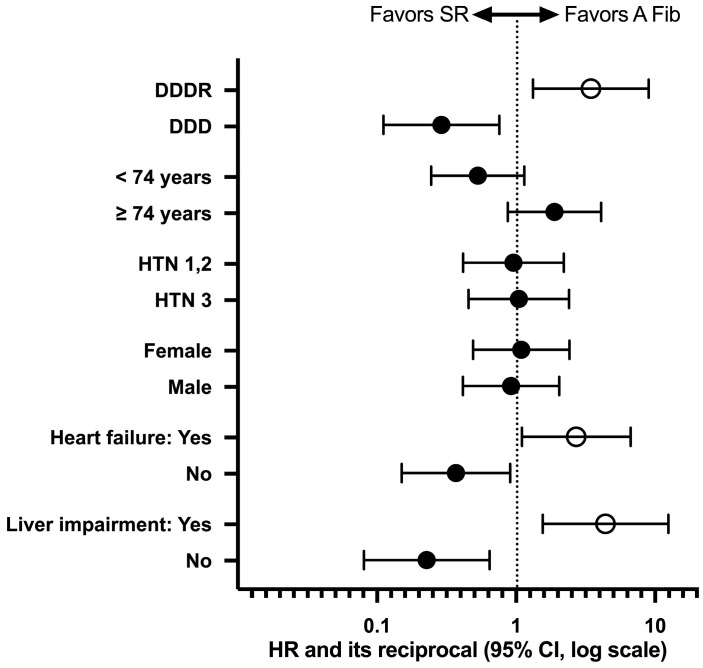
Hazard ratio and its reciprocal with 95% confidence interval (CI) for patients included in our study. The empty circles represent a statistically significant difference.

The age of the patients did not influence the occurrence of A Fib, in the sense that older patients had an A Fib occurrence rate at the 9-month follow-up of 18% vs. without A Fib, where the rate was only 9.756%, but without any statistical difference (HR = 1.888, CI 0.8726 to 4.084, 0.1190, Figure 3B). Regarding hypertensive patients, there were no significant differences, the rate of A Fib occurrence being in patients with hypertension 3rd grade (HTN 3) of 13.934% vs. patients with lower grades of hypertension (HTN 1,2) where the rate of A Fib occurrence was approximately equal, 13.333% (HR = 1.045, CI 0.4538 to 2.405, *p* = 0.9168, Figure 3C). Also, the gender of the patients did not significantly influence the occurrence of atrial fibrillation in patients wearing pacemakers, the rate of A Fib occurrence in women being 14.705% and in men being 14.035% at the 9-month follow-up (HR= 1.088, CI 0.4895 to 2.417, *p* = 0.8304, Figure 3D). On the other hand, patients with advanced heart failure (NYHA III) had a much higher rate of A Fib (26.086%) compared to patients with NYHA I/II heart failure, who had a much lower rate (10.294%) at the 9-month follow-up (HR = 2.708, CI = 1.101 to 6.656, *p* = 0.0068, Figure 3E). Finally, the existence of hepatic impairment (HI) was a predictor for the occurrence of A Fib, meaning that patients with HI had an A Fib occurrence rate of 35.294% vs. 9.459% in patients without HI (HR = 4.398, CI 1.554 to 12.45, *p* < 0.0001, Figure 3F). Summarizing this analysis, we identified as predictors for the occurrence of atrial fibrillation in patients with cardiac pacemakers the DDDR pacing mode, NYHA class III heart failure, and the presence of hepatic impairment (HI). For all these factors, we have shown in Figure 4 the hazard ratio and its reciprocal, as well as the 95% CI of the ratio.

**Figure 5:**
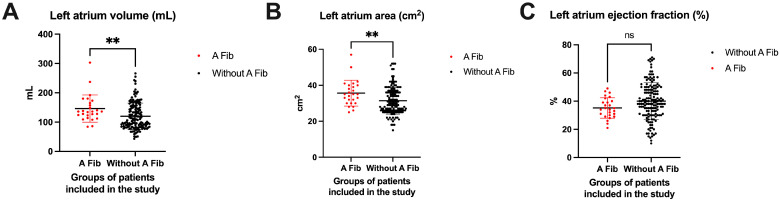
The assessment of echocardiographic parameters of the left atrium. (**A**) Volume, (**B**) area, and (**C**) ejection fraction. ** *p* < 0.01.

The analysis of echocardiographic parameters of the left atrium revealed that a larger volume of the left atrium was associated with the occurrence of atrial fibrillation. Thus, in the group of patients in whom the occurrence of atrial fibrillation was identified at follow-up, a mean volume of 146.1 ± 46.29 mL was noted at baseline, compared to patients who did not develop atrial fibrillation, where the mean volume at baseline was 119.7 ± 45.07 (*p* = 0.0065). Also, statistically significant differences were found in the case of patients who at baseline presented a larger left atrial area (35.65 ± 7.155 cm^2^) compared to patients who had a much smaller area (31.44 ± 7.428 cm^2^) at baseline and who at follow-up did not present atrial fibrillation at all (*p* = 0.0078). It was very interesting that there were no statistically significant differences in the left atrial ejection fraction at baseline between the two groups of patients (*p* = 0.0078), although the A Fib group had a lower mean ejection fraction (35.19 ± 7.239%) compared to the group without A Fib (40.06 ± 13.07%). All these observations are highlighted in Figure 5.

**Figure 6:**
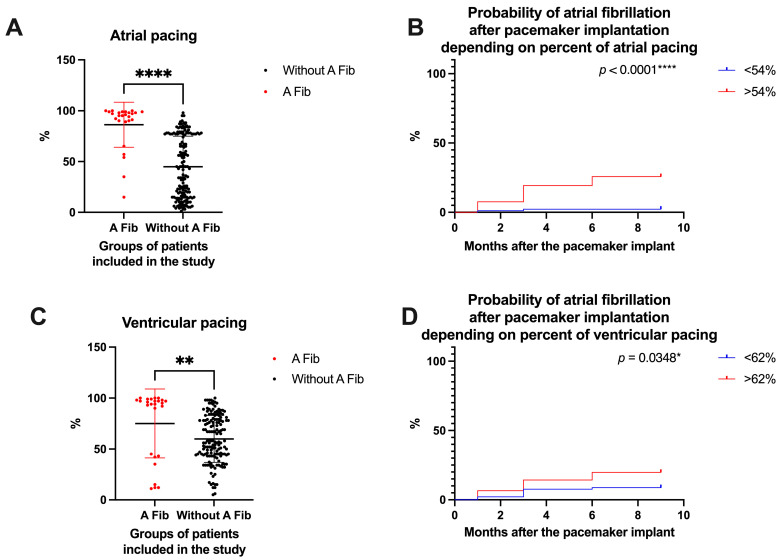
(**A**) Percent of atrial pacing (AP), (**B**) probability of atrial fibrillation depending on AP, (**C**) percent of ventricular pacing (VP), and (**D**) probability of atrial fibrillation depending on VP. **** *p* < 0.0001; ** *p* < 0.01; * *p* < 0.05.

The percentage of atrial/ventricular pacing was another important analysis in our study, especially since there are not many studies that have analyzed these parameters. Patients in the A Fib group presented a higher percentage of atrial pacing at the 9-month follow-up (86.23 ± 22.19%) compared to patients in the group without A Fib (44.92 ± 29.99%, *p* < 0.0001, Figure 6A). On the other hand, in the group of patients with A Fib, those with a higher percentage of atrial pacing had an A Fib occurrence rate at the 9-month follow-up of 25.806% compared to those with a low percentage of atrial pacing who had an A Fib occurrence rate of only 2.247% (HR = 12.38, CI 5.739 to 26.71, *p* < 0.0001, Figure 6B). Regarding the percentage of ventricular pacing at the 9-month follow-up, the observations were almost similar. First, patients in the A Fib group presented a high percentage of ventricular pacing at the 9-month follow-up (75.04 ± 33.86%) compared to patients in the group without A Fib (59.94 ± 23.12%, *p* = 0.0047, Figure 6C). And in this case, in the group of patients with A Fib, those with a higher percentage of ventricular pacing recorded an A Fib occurrence rate at the 9-month follow-up of 19.780% compared to those with a low percentage of ventricular pacing who recorded an A Fib occurrence rate of only 8.791% (HR = 2.338, CI 1.084 to 5.044, *p* = 0.0348, Figure 6D).

## 4. Discussion

In this study, we confirmed that classical factors such as advanced heart failure or large left atrial volumes and areas, as well as much less studied factors such as the percentage of atrial/ventricular pacing or the presence of hepatic impairment, are predictors of the occurrence of atrial fibrillation in patients with cardiac pacemakers. It is well known that A Fib is one of the most common cardiac arrhythmias, which can lead to a decreased efficiency of the heart’s pumping capacity and an increased risk of stroke [19]. The occurrence of atrial fibrillation in patients with pacemakers represents a particular clinical challenge, especially in the context in which these patients usually have significant comorbidities that further complicate their medical management [20]. When rhythm disturbances are observed in people with hepatic impairment, the complexity of treatment increases, given that the liver is crucial in the metabolism of many drugs used to control and prevent arrhythmias on the one hand, and on the other hand, the administration of anticoagulant treatment can further complicate the situation of these patients. Hepatic impairment can alter the dynamics of the action of these drugs, potentially increasing the risk of toxicity and harmful drug interactions. In the management of these patients, an interdisciplinary approach is required, which should include careful monitoring of liver function, adaptation of the therapeutic scheme, and optimization of the use of pacemaker technology to promptly identify arrhythmic episodes and adjust the treatment accordingly. In both Europe and the USA, the rate of newly diagnosed atrial fibrillation in patients with pacemakers is quite high according to studies published to date, reaching approximately 20–50% [21,22,23,24,25]. Older studies that analyzed the occurrence of atrial fibrillation in the general population, such as the Framingham Heart Study, identified a rate of approximately 0.5%, but the diagnostic method was generally diagnosed by ECG or Holter monitoring, but these methods are not useful in the early detection of A Fib due to the short monitoring period [26]. CIEDs, including pacemakers, can record atrial arrhythmia at any time and are extremely useful in the early diagnosis of atrial fibrillation [21]. Furthermore, in the current era of artificial intelligence (AI), equipping pacemakers with AI tools can improve the detection rate of A fib [27]. It is worth mentioning that a recent study that included over 22,000 patients showed that the incidence of device-detected atrial tachyarrhythmia is approximately 23%, which was also reported in the previously cited studies, but the incidence of thromboembolic events was approximately 3.7%, and over two-thirds of these patients maintained sinus rhythm, which makes their identification even more difficult [28].

Cardiac device programming should be considered as a predictor of atrial fibrillation in patients wearing such devices, in addition to known risk factors such as age, sex, hypertension, or pharmacological therapy [29,30]. A positive correlation has been demonstrated between the incidence of atrial fibrillation and the percentage of right ventricular pacing, but studies have also shown a link between the percentage of atrial pacing and atrial fibrillation [31,32]. A meta-analysis of 1507 patients from four studies that included patients with pacemakers found that right atrial pacing was associated with a higher incidence of atrial fibrillation in patients with sick sinus syndrome [33]. However, a sub-analysis of a Danish multicenter randomized trial that examined right atrial pacing versus bicameral pacing in sick sinus syndrome found no association between right atrial pacing and atrial fibrillation in patients with sick sinus syndrome [34]. Another parameter analyzed was the Heart Rate Score (HRSc), which has been shown to be the primary predictor of atrial tachycardia in patients with pacemakers, regardless of age, gender, percentage of ventricular pacing, or rate response programming [35,36].

The assessment of embolic risk associated with newly diagnosed atrial fibrillation in patients with pacemakers has been an important objective of several studies. In the literature, the prevalence of embolic events associated with atrial fibrillation has ranged from 1.5% to 23.5% [37]. The overall rate of embolic events in one cohort was 2.1% compared to a reported stroke rate of 2.7% in the general population [38]. The temporal dissociation of atrial tachyarrhythmic events detected in patients with pacemakers and embolic events makes it difficult to establish a clinically relevant threshold for the diagnosis of newly diagnosed atrial fibrillation in patients with cardiac pacemakers. Previous studies have shown that atrial tachyarrhythmic events rarely occur simultaneously with embolic events but may occur before or after embolic events, by weeks or even months [28]. However, strong evidence indicates that DDAT is significantly associated with increased risk, regardless of the causal mechanism [28]. In 8181 patients with previously undiagnosed atrial tachyarrhythmia events with diagnostic thresholds ranging from 30 s to 14 min, the overall risk of embolic events was found to be twofold [28]. The risk was lower when a threshold of less than 1 min was used (RR, 1.77; *p* = 0.01) and was not significantly associated with an increased risk of embolic events when a threshold of ≥5 min was used (RR, 3.86; *p* = 0.01) [28]. This may suggest a dose-dependent relationship between the duration of atrial tachyarrhythmias and the risk of embolic events. In a recent sub-analysis of the ASSERT (Asymptomatic Atrial Fibrillation and Stroke Evaluation in Pacemaker Patients and the Atrial Fibrillation Reduction Atrial Pacing Trial) study by Van Gelder et al., it was found that a pacemaker-detected atrial tachyarrhythmia lasting more than 24 h was most frequently associated with embolic events, and patients with pacemaker-detected atrial tachyarrhythmias lasting more than 6 min to 24 h had a risk of embolic events similar to that of patients without these rhythm disturbances [9]. However, data on longer episodes are lacking in large retrospective studies. Separately, there are data showing that short duration of atrial tachyarrhythmia events, when carefully considered, is not correlated with a higher risk of clinical events (as in the RATE [Register of Atrial Tachycardia and Fibrillation Episodes] registry) [38]. However, it is unclear what minimum threshold of pacemaker-detected atrial tachyarrhythmia duration could stratify the risk of thromboembolic events, and this remains to be studied.

The management of patients with atrial fibrillation and hepatic impairment should be performed with caution, even when they use pacemakers, because the choice of antiarrhythmic or anticoagulant drugs should be adjusted for patients with known liver disease, and some drugs may require more intensive monitoring or reduced doses. First, liver disease creates an inflammatory state that triggers the de novo development of atrial fibrillation [39]. Active liver disease induces various proinflammatory cytokines, which can infiltrate the heart, leading to infiltration and inflammation of immune cells—key elements of the liver–heart axis that could culminate in atrial fibrillation [40]. A recent study validated a set of five differentially expressed and co-upregulated genes (DEGs) (DGAT1, AMOT, PDE11A, TYMS, and TMEM98) that were elevated in patients with atrial fibrillation, liver disease, and atrial fibrillation associated with liver disease compared to healthy controls [40]. Notably, the latter four genes were specifically upregulated in patients with liver failure and concomitant atrial fibrillation. Another link implicated in the pathophysiology of atrial fibrillation and liver disease is mitochondrial dysfunction and systemic inflammation [41]. Under metabolic stress, the production of reactive oxygen species (ROS) increases in mitochondria, and once ROS levels exceed the neutralization capacity of the own antioxidant system, they damage the mitochondrial membrane and mitochondrial DNA, inducing inflammation in the liver and causing mitochondrial dysfunction through nuclear factor κB (NF-κB) or through other factors such as the nucleotide-binding oligomerization domain-like receptor family pyrin domain-containing 3 (NLRP 3) [41]. When cardiomyocyte mitochondrial dysfunction occurs, ATP production decreases, leading to the opening of ATP-sensitive potassium channels at the membrane level, thus causing the slowing of cardiomyocyte electrical impulse conduction, increased inequality, and ease of refractoriness, which induces atrial fibrillation [42]. In addition, myocardial mitochondria store a large amount of Ca^2+^ and when mitochondrial dysfunction occurs, this balance is altered [43]. In addition to all these pathophysiological links, which also explain the results of our study, a particularly important problem is related to the administration of anticoagulant treatment in patients with liver damage and atrial fibrillation [20]. Although patients with atrial fibrillation and liver disease benefit from anticoagulation for stroke prevention, treatment with oral anticoagulants is challenging in patients with abnormal liver function due to the combination of intrinsic coagulopathy and increased bleeding risk [44]. Given that direct oral anticoagulants, compared to warfarin, are less dependent on the liver for elimination (20% for dabigatran, 50% for edoxaban, 65% for rivaroxaban, and 75% for apixaban, compared to 100% for warfarin), many studies have shown that they could be a suitable alternative to warfarin for patients with advanced liver disease [44,45,46,47,48,49].

### Limitations of Our Study

This study has several limitations that should be considered when interpreting the results. First, it is a retrospective cohort study conducted in a single center, which means that the results may not be generalizable to other practice settings. In addition, the sample size and mean follow-up period are small, which may limit the statistical power of the study and make it difficult to draw definitive conclusions. Another limitation is that the default pacemaker settings for A Fib detection were slightly different between different models, although devices from only two manufacturers were included. As a result, the findings may not be directly applicable to all real-life decisions and patient management.

## 5. Conclusions

The importance of pacemakers in detecting subclinical episodes of atrial fibrillation remains very important for the prevention of embolic events in these patients. Moreover, in the association of risk factors for the occurrence of atrial fibrillation, special attention should be paid to these patients in the early identification of atrial tachyarrhythmias. Hepatic impairment may be a risk factor for the occurrence of atrial fibrillation in patients with pacemakers, but it can also create significant problems in the prevention of stroke.

## Figures and Tables

**Table 1:** Baseline features of the patients according to the end point of our study, the presence or absence of atrial fibrillation at the 9-month follow-up.

Variable Baseline Features		Atrial Fibrillation	Without Atrial Fibrillation	*p*-Value
Gender	Male	16 (14.04%)	98 (85.96%)	>0.9999
	Female	10 (14.71%)	58 (85.29%)	
Age (years)		77.31 ± 7.092	72.4 ± 8.015	0.0038 ^#^
Hypertension	1/2	8 (13.33%)	52 (86.67%)	>0.9999
	3	18 (14.75%)	105 (85.25%)	
Heart failure	NYHA I/II	14 (10.29%)	122 (89.71%)	0.0135 *
	NYHA III/IV	12 (26.09%)	34 (73.91%)	
Pacing mode	DDD	14 (10.14%)	126 (89.86%)	0.0112 *
	DDDR	12 (27.27%)	32 (72.73%)	
QRS duration (ms)		147.4 ± 12.99	144.5 ± 8.501	0.481
Hepatic impairment	Yes	12 (35.29%)	22 (64.71%)	0.0004 *
	No	14 (9.46%)	134 (90.54%)	
Echo parameters	LVEF (%)	52.96 ± 4.306	54.27 ± 2.796	0.3309

^#,^* *p* < 0.05, ^#^ T test. * Fisher’s exact test.

## Data Availability

Data are contained within the article or available upon request from the corresponding authors.

## Appendix A

DDD/DDDR = A letter code named NBG code, describing the function of the various pacing systems. DDDR modes include pacing and sensing capabilities in both the ventricles and atria: dual-chamber (atrial and ventricular) stimulation; dual-chamber (atrial and ventricular) sensing; dual response to detection (triggering and inhibition); R-rate responsive (can accelerate heart rhythm according to need).
